# Supplementary material for: Mobile fluxons as coherent probes of periodic pinning in superconductors
Source: Sci Rep. 2017 Oct 23;7:13740. doi: 10.1038/s41598-017-14232-z (PMC5653780; doi:10.1038/s41598-017-14232-z)
Supplement: Supplementary file 1 — Supplementary Information [file 41598_2017_14232_MOESM1_ESM.pdf]

Supplementary information for the manuscript

Mobile fluxons as coherent probes of periodic pinning in superconductors

Oleksandr V. Dobrovolskiy[1,2], Michael Huth[1], Valerij A. Shklovskij[2], and Ruslan V. Vovk[2]

[1]*Physikalisches Institut, Goethe University, Frankfurt am Main, 60438, Germany*

[2]*Physics Department, V. Karazin National University, Kharkiv, 61022, Ukraine*

Frequency dependence of the power absorption

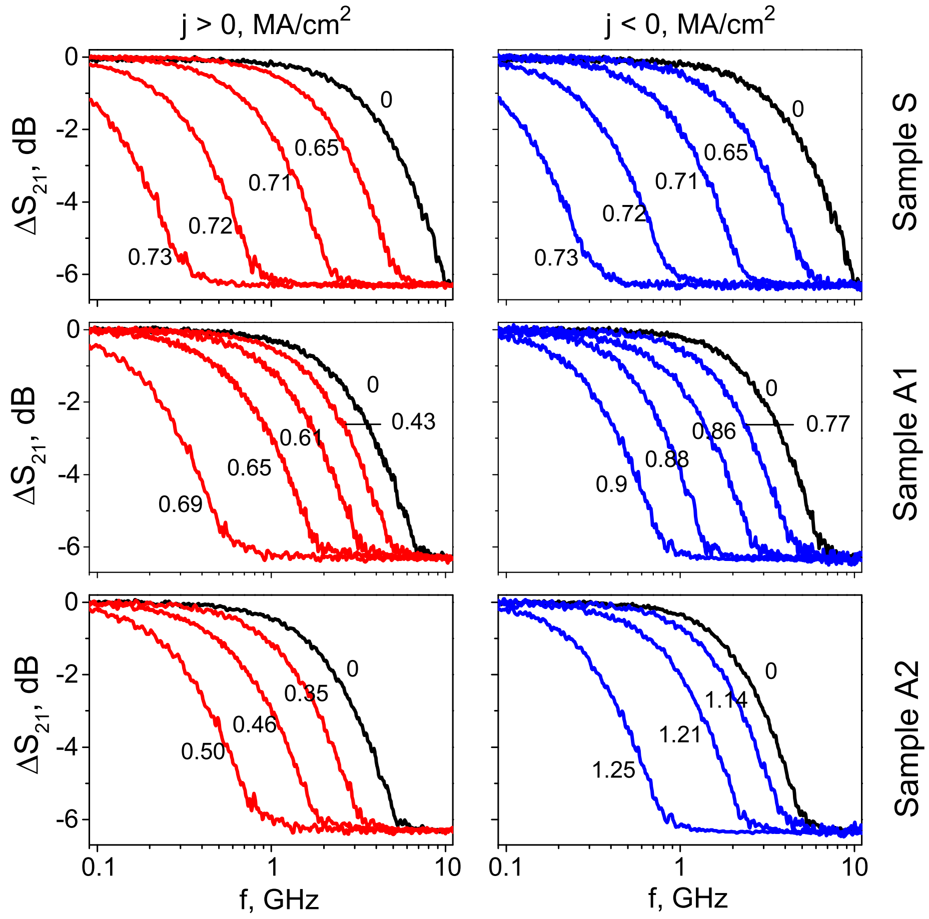

Figure 1. Frequency dependence of the relative change of the absolute value of the forward transmission coefficient $\Delta S_{21}(f)$ of all samples at positive (left column, red curves) and negative (right column, blue curves) dc densities, as indicated, at $T = 0.3T_c$ and the fundamental matching field $H_m = 7.2$ mT. At dc biases of the positive polarity, the dc Lorentz force is directed against the gentle groove slope.

Mechanistic scenario for mode-locking fringes to appear

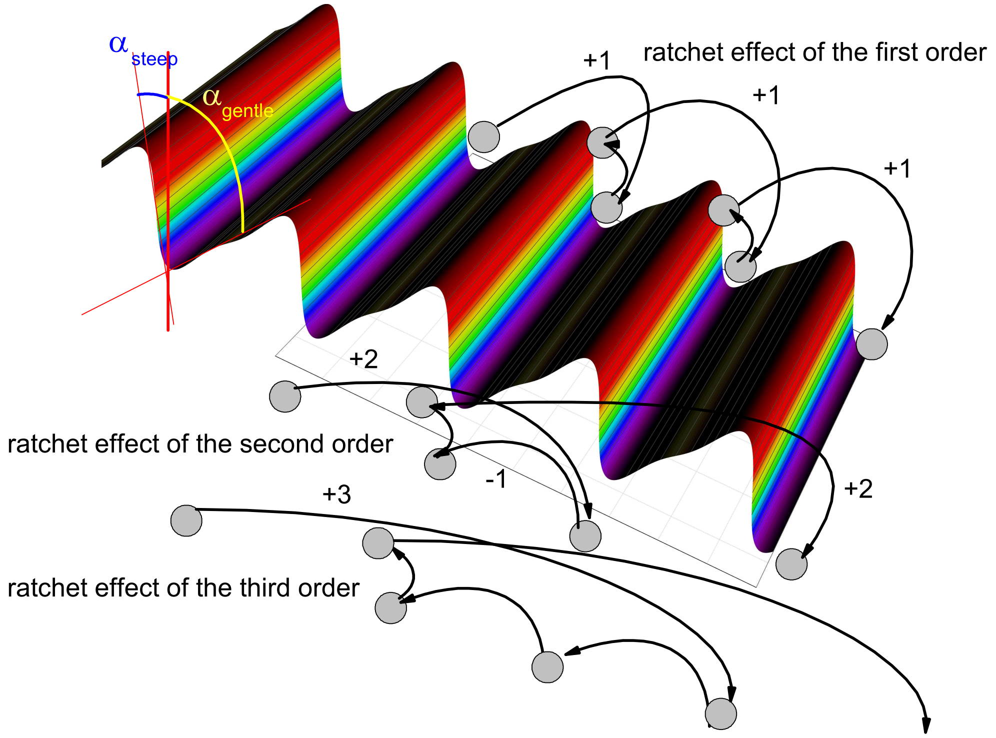

Figure 2. In the ratchet effect of the first order, during one ac period a vortex (grey circle) overcomes the right barrier during one half ac period, but the ac amplitude is not enough to let the vortex overcome the left barrier during the other half of the ac period. With increasing ac amplitude the vortex can overcome two barriers to right and either one (shown in the sketch) or no (not shown in the sketch) barrier to the left and so on. For the ratchet effect of the third order, only a vortex overcoming three barriers to the right and no barrier to the left is shown. Each order of the ratchet effect corresponds to a peak or dip (depending on the polarity of the applied dc bias) in the dependence of the dc voltage on the ac amplitude. The angles α_{steep} and α_{gentle} indicate the angles under which the slopes of the asymmetric WPP are tilted with respect to the vertical axis which stands for the pinning potential $U(x)$. For the orientation of the steep and gentle slopes shown in the sketch, the condition of the effective symmetrization $\alpha_{\text{steep}} = \alpha_{\text{gentle}}$ will be realized at some dc bias value resulting in a tilt of the pinning potential to the left. In reality, however, the assumption that the slopes of the potential can be modelled as planes is too crude. But here the angles α are introduced just for an obvious explanation how the internal asymmetry of the ratchet WPP can be "compensated" by the tilt induced by the dc bias. In the linear approximation, the physical meaning of these angles is the pinning forces for the respective groove slopes.

Determination of the pinning potential

The procedure of the determination of the coordinate dependence of the pinning potential is based on the Gittleman-Rosenblum (GR) model[1] developed for zero temperature and generalized for the case of arbitrary dc and ac bias values[2]. In this model, the equation of motion for a vortex moving with velocity $v(t)$ in some pinning potential under the action of superimposed dc and high-frequency ac currents reads

$$\eta v(t) = F(t) + F_p, \qquad (1)$$

where η is the vortex viscosity, $v(t)$ is the vortex velocity, and $F(t) = \frac{\Phi_0}{c}(j + j^{mw}(t))$ is the Lorentz force, where j is the dc current density and $j^{mw}(t) \equiv j^{mw} \exp 2\pi i f t$ with j^{mw} is the amplitude of the microwave current. In Eq. (1), $F_p = -dU(x)/dx$ is the pinning force and $U(x)$ is the sought-for pinning potential with the depth U_0 and period a. Henceforth we scale j to j_d, U to U_0 and x to a. Accordingly, the pinning potential is related[2,3] to the dc current-induced reduction of the depinning frequency via

$$U(x) = \int_0^x dx_0 \frac{j(x_0)}{j_d}, \qquad (2)$$

where x_0 is the rest point of the vortex in the tilted pinning potential in the absence of the mw current. Further, the function $j(x_0)$ is the inverse function to $x_0(j)$ given by

$$x_0(j) = \int_0^j \frac{dj'}{f_d(j')/f_0}, \qquad (3)$$

where $f_d(j')/f_0$ should be deduced from the experimental data.

We approximated the reduction of the depinning frequency for both dc polarities for sample S by the following expression

$$f_d/f_0 = [1 - (j/j_d)^2]^{1/2}, \qquad |j| < |j_d|. \qquad (4)$$

For the gentle-slope direction of the asymmetric potential of sample A1 probed by the positive halfwave of the ac current, the fit reads

$$f_d/f_0 = [1 - (j/j_d)^{3/2}]^{1/2}, \quad 0 < j < j_d^+, \qquad (5)$$

while for its steep-slope direction probed by the positive halfwave of the ac current, the dependence reads

$$f_d/f_0 = [1 - (j/j_d)^3]^{1/2}, \quad -j_d^- < -j < 0. \qquad (6)$$

For the gentle-slope direction of the asymmetric potential of sample A2 probed by the positive halfwave of the ac current, the reduction of the depinning frequency can be fitted to

$$f_d/f_0 = [1-(j/j_d)^{3/2}]^{2/3}, \quad 0 < j < j_d^+, \tag{7}$$

while for its steep-slope direction probed by the positive halfwave of the ac current, the approximation reads

$$f_d/f_0 = [1-(j/j_d)^4]^{1/4}, \quad -j_d^- < -j < 0. \tag{8}$$

The deduced dependence $U(x)/U_0$ in sample S is symmetric with respect to the line $x/a = 0.5$ and it fits very well to

$$U(x)/U_0 = [1-\cos 2\pi x]/2. \tag{9}$$

The deduced dependences $U(x)/U_0$ for samples A1 and A2 are asymmetric with respect to the line $x/a = 0.5$. For sample A1 $U(x)/U_0$ exhibits a maximum at $x \approx 0.44$ and it can be satisfactory fitted to

$$U(x)/U_0 = [(1-\cos 2\pi x) + 0.13(1-\sin 4\pi x)/2]/2. \tag{10}$$

The deduced dependence $U(x)/U_0$ for sample A2 is most strongly asymmetric. The curve $U(x)/U_0$ has a maximum at $x \approx 0.32$ and it can be fitted rather well to

$$U(x)/U_0 = [(1-\cos 2\pi x) + 0.5(1-\sin 4\pi x)/2]/2. \tag{11}$$

while the AFM profile suggests that also it can be fitted satisfactory to the same expression.

References

1. Gittleman, J. I. & Rosenblum, B. Radio-frequency resistance in the mixed state for subcritical currents. *Phys. Rev. Lett.* **16**, 734–736 (1966). URL http://link.aps.org/doi/10.1103/PhysRevLett.16.734. DOI 10.1103/PhysRevLett.16.734.
2. Shklovskij, V. A. & Dobrovolskiy, O. V. *Microwave Absorption by Vortices in Superconductors with a Washboard Pinning Potential*, chap. 11, 263–288 (InTech, Rijeka, 2012).
3. Shklovskij, V. A. Determination of coordinate dependence of the washboard pinning potential from the dynamic experiment with vortices. In *Procedings of the Fifth International Conference on Mathematical Modeling and Computer Simulation of Materials Technologies MMT-2008, Ariel, Israel* (2008).
